# Full Genome Sequence of Giant Panda Rotavirus Strain CH-1

**DOI:** 10.1128/genomeA.00241-12

**Published:** 2013-02-21

**Authors:** Ling Guo, Qigui Yan, Shaolin Yang, Chengdong Wang, Shijie Chen, Xiaonong Yang, Rong Hou, Zifang Quan, Zhongxiang Hao

**Affiliations:** a College of Veterinary Medicine, Sichuan Agricultural University, Ya’an, China; b Key Laboratory of Animal Disease and Human Health of Sichuan Province, Sichuan Agricultural University, Ya’an, China; c Conservation and Research Center for the Giant Panda, Ya’an, Sichuan, China; d Sichuan Entry-Exit Inspection and Quarantine Bureau, Chengdu, China; e Southwest University for Nationalities, Chengdu, Sichuan, China; f Chengdu Research Base of Giant Panda Breeding, Chengdu, China

## Abstract

We report here the complete genomic sequence of the giant panda rotavirus strain CH-1. This work is the first to document the complete genomic sequence (segments 1 to 11) of the CH-1 strain, which offers an effective platform for providing authentic research experiences to novice scientists.

## GENOME ANNOUNCEMENT

Rotaviruses (RVs) are members of the family *Reoviridae* and cause severe diarrheal illness in the young of various animal species (1). Since its discovery in 1963, rotavirus has been recognized as the leading cause of infectious gastroenteritis worldwide (2). Rotavirus contains 11 segments (Seg-1 to Seg-11) of double-stranded RNA. These segments are responsible for the six structural viral proteins (VP) (VP1, VP2, VP3, VP4, VP6, and VP7) and five nonstructural proteins (NSP) (NSP1 to NSP5) that comprise the complex triple-layered capsid structure (2–5). Worldwide, there has been no report of the full genomic sequence of the giant panda rotavirus strain. It is necessary to acquire and analyze the sequence of the complete genome of a giant panda rotavirus strain to study its molecular epidemiology.

Here, we report a rotavirus strain, CH-1, isolated from fecal samples of a giant panda with acute diarrhea symptoms, in the Chengdu Research Base of Giant Panda Breeding (CPB) (6). Viral double-stranded RNA (dsRNA) from cells infected with CH-1 was isolated using TRIzol (Invitrogen) as described by Attoui et al. (7). Eleven pairs of oligonucleotide primers were used to amplify the different regions of the CH-1 genome. The PCR products were purified, then cloned into the pMD19-T vector (TaKaRa, Japan). The cloned inserts were then sequenced by Invitrogen to determine the full genomic sequence of CH-1.

The sizes (in base pairs [bp]) of segments 1 to 11 from CH-1 are 3,267, 2,673, 2,508, 2,362, 1,194, 981, 1,461, 954, 942, 528, and 594, respectively. The amino acid (aa) lengths of the six structural proteins of CH-1 (VP1 to VP4, VP6, and VP7) are 1,088, 890, 835, 776, 397, and 326, respectively. The amino acid lengths of the five nonstructural proteins of CH-1(NSP1 to NSP5) are 486, 317, 313, 175, and 197, respectively. Our genetic analysis demonstrated that the genomic constellation of G1P[7] rotavirus was G1-P[7]-I5-R1-C1-M1-A1-N1-T1-E1-H1.

The data presented here are the first to report the complete sequence of a giant panda rotavirus strain isolated in China and represent the only first complete sequence of giant panda rotavirus in the world. These data will facilitate future investigations of the molecular characteristics and geographic origins of giant panda rotavirus strains from China, as well as from other countries.

### Nucleotide sequence accession numbers. 

The full genomic sequence of the giant panda rotavirus strain CH-1 was deposited in GenBank. The accession no. HQ641297, HQ641294 to HQ641296, GU188283, GU188284, GU205762, GU188281, GU329525, GU188282, and GU329526 correspond to CH-1 segments 1 through 11, respectively.
